# An accurate circuit-based description of retinal ganglion cell computation

**DOI:** 10.1186/1471-2202-16-S1-O9

**Published:** 2015-12-18

**Authors:** Yuwei Cui, Yanbin V Wang, Jonathan B Demb, Daniel A Butts

**Affiliations:** 1 Department of Biology and Program in Neuroscience and Cognitive Science, University of Maryland, College Park, MD 20742, USA; 2 Department of Ophthalmology and Visual Science and Department of Cellular and Molecular Physiology, Yale University, New Haven, CT 06511 USA

## 

Visual processing depends on computations performed by complex neural circuits. Although the circuitry in the retina has been extensively characterized, common "functional" models of how ganglion cell spike trains represent visual stimuli typically rely on linear descriptions of their receptive field [1]. Different types of nonlinear models have offered improvements in spike train prediction, but such improvements are often incremental, and in most cases not linked to known elements of the retinal circuit.

Here, we describe a new nonlinear model framework designed to represent key elements of the retinal circuit, which can predict recorded retinal ganglion cell spike trains with high temporal precision. We used recordings of both synaptic currents (via voltage-clamp recordings) and spike (via loose patch recordings) from the same ON Alpha ganglion cells in the mouse retina in order to build a two-stage nonlinear model. This model describes ganglion cell computation as sums and products of excitatory and inhibitory inputs [2]. Model parameters were estimated based on either intracellular or spike train data using a maximum-likelihood framework.

We found that excitatory synaptic currents to the ganglion cell are well described by an excitatory input combined with divisive suppression, both elements described by LN models fit to intracellular data. Using stimuli with center-surround structure, we demonstrate that this divisive suppression arises from the surround, and is the likely result of presynaptic inhibition mediated by amacrine cells [3], rather than synaptic depression [4]. We then extended this nonlinear model of synaptic currents to explain spike response of the ganglion cell by incorporating a spiking nonlinearity with spike refractoriness. All model parameters could be fit using the spike trains alone, resulting in a prediction of the excitatory currents that closely matched the models fit directly to the currents.

The resulting model had unprecedented ability to predict both synaptic current and spike trains (with >90% of the explainable variance) at one millisecond resolution on cross-validation datasets, capturing both fast transient responses in synaptic current, as well as the high precision of spike train responses. Furthermore, the model output automatically "adapted" to contrast, and could predict the responses across contrast levels with similar accuracy without any change in model parameters. Notably, the nonlinear structure of the model was particular to ON Alpha ganglion cells, and other retinal ganglion cell types had distinct computational structures, likely corresponding to different underlying connectivity within the retina governing their processing of vision.

Thus, by targeting a nonlinear model based on the specific computations performed by retinal circuit elements, we uncovered an extremely accurate description of retinal processing, and identified two-stage computational properties that can be linked to elements of the retinal circuit. In addition to providing an accurate description of ON Alpha cells, such computational framework also sets a foundation for understanding the different roles of the ~20 ganglion cell types that comprise the input to the rest of the visual system.
